# Reducing antimicrobial overuse through targeted therapy for patients with community-acquired pneumonia: a study protocol for a cluster-randomized factorial controlled trial (CARE-CAP)

**DOI:** 10.1186/s13063-023-07615-3

**Published:** 2023-09-16

**Authors:** Abhishek Deshpande, Ramara Walker, Rebecca Schulte, Andrea M. Pallotta, Larisa G. Tereshchenko, Bo Hu, Sameer S. Kadri, Michael Klompas, Michael B. Rothberg

**Affiliations:** 1 Center for Value-Based Care Research, Primary Care Institute, Cleveland Clinic, Cleveland, OH USA; 2 Department of Infectious Disease, Cleveland Clinic, Cleveland, OH USA; 3 Department of Pharmacy, Cleveland Clinic, Cleveland, OH USA; 4 Department of Quantitative Health Sciences, Cleveland Clinic, Cleveland, OH USA; 5 Critical Care Medicine Department, National Institutes of Health Clinical Center, Bethesda, MD USA; 6 Department of Population Medicine, Harvard Medical School and Harvard Pilgrim Health Care Institute, Boston, MA USA; 7 Department of Medicine, Brigham and Women’s Hospital, Boston, MA USA

**Keywords:** Community-acquired pneumonia, Rapid diagnostic testing, Antimicrobial stewardship, Extended-spectrum antibiotics

## Abstract

**Background:**

Community-acquired pneumonia (CAP) is a significant public health concern and a leading cause of hospitalization and inpatient antimicrobial use in the USA. However, determining the etiologic pathogen is challenging because traditional culture methods are slow and insensitive, leading to prolonged empiric therapy with extended-spectrum antibiotics (ESA) that contributes to increased hospital length of stay, and antimicrobial resistance. Two potential ways to reduce the exposure to ESA are (a) rapid diagnostic assays that can provide accurate results within hours, obviating the need for empiric therapy, and (b) de-escalation following negative bacterial cultures in clinically stable patients.

**Methods:**

We will conduct a large pragmatic 2 × 2 factorial cluster-randomized controlled trial across 12 hospitals in the Cleveland Clinic Health System that will test these two approaches to reducing the use of ESA in adult patients (age ≥ 18 years) with CAP. We will enroll over 12,000 patients and evaluate the independent and combined effects of routine use of rapid diagnostic testing at admission and pharmacist-led de-escalation after 48 h for clinically stable patients with negative cultures vs usual care. We hypothesize that both approaches will reduce days on ESA. Our primary outcome is the duration of exposure to ESA therapy, a key driver of antimicrobial resistance. Secondary outcomes include detection of respiratory viruses, treatment with anti-viral medications, positive pneumococcal urinary antigen test, de-escalation by 72 h from admission, re-escalation to ESA after de-escalation, total duration of any antibiotic, 14-day in-hospital mortality, intensive care unit transfer after admission, healthcare-associated *C. difficile* infection, acute kidney injury, total inpatient cost, and hospital length-of-stay.

**Discussion:**

Our study aims to determine whether identifying an etiological agent early and pharmacist-led de-escalation (calling attention to negative cultures) can safely reduce the use of ESA in patients with CAP. If successful, our findings should lead to better antimicrobial stewardship, as well as improved patient outcomes and reduced healthcare costs. Our findings may also inform clinical guidelines on the optimal management of CAP.

**Trial registration:**

ClinicalTrials.gov NCT05568654. Registered on October 4, 2022.

**Supplementary Information:**

The online version contains supplementary material available at 10.1186/s13063-023-07615-3.

## Introduction

### Background and rationale

Community-acquired pneumonia (CAP) is a significant cause of morbidity and mortality, resulting in > 1.7 million adult hospitalizations annually, making it the second most common cause of hospitalization in the USA [1–3]. One contributing factor to the overuse of extended-spectrum antibiotics (ESA) for CAP is that the causative pathogen is unknown in nearly half of cases, and initial treatment is almost always empiric [4, 5]. Guidelines of the American Thoracic Society and Infectious Diseases Society of America (ATS/IDSA) recommend limiting ESA to inpatients with risk factors for resistant pathogens, but many inpatients with CAP receive ESA, often throughout their hospital stay [6–8].

An accurate pathogenic diagnosis can contribute to antimicrobial stewardship in 2 ways: (1) by allowing for initial narrow-spectrum therapy and (2) by providing confidence when de-escalating therapy following negative cultures. However, without a diagnostic test, many clinicians are uncomfortable with de-escalation, fearing an undiagnosed resistant pathogen could cause treatment failure. Rapid molecular diagnostic assays have the potential to identify causative pathogens, but they are rarely part of the diagnostic work-up, and results are often ignored [9, 10]. In a national sample of patients with CAP from 177 US hospitals, we found that only 16% had a pneumococcal urinary antigen testing (UAT) performed and < 25% were tested for respiratory viruses [11, 12]. Both the ATS/IDSA CAP guidelines and the antimicrobial stewardship implementation guidelines recommend routine testing of inpatients for influenza during influenza activity; neither one recommends routine UAT testing [6, 13]. Both sets of recommendations are based on limited evidence and it is not known whether widespread testing for viruses would reduce antibiotic use. In a multi-hospital dataset, we found that patients who tested positive for respiratory viruses received 0.8 fewer days of antibiotics, and patients who tested positive with UAT were twice as likely to have their ESA de-escalated by hospital day 3 [11, 12]. There is therefore a need for large, randomized trials that can evaluate the impact of initial diagnostic testing on antibiotic prescribing and patient outcomes.

De-escalation following negative bacterial cultures is another antimicrobial stewardship target. While most de-escalation follows the identification of a susceptible pathogen, the IDSA/ATS guidelines also recommend de-escalation to a narrower spectrum at 48 h if cultures are negative [6]. However, this recommendation is based on observational studies. In our study of 164 US hospitals, we found that de-escalation practices following negative cultures varied widely by hospital, and no hospital de-escalated even 40% of eligible patients [14]. De-escalation was associated with shorter length of stay and lower rates of inpatient mortality. A hospital-wide pharmacist-led effort to encourage physicians to follow the ATS/IDSA guidelines and de-escalate after 48 h could help overcome fears about resistant pathogens and treatment failure.

### Objectives and study aims

This large, multicenter 2 × 2 factorial cluster-randomized controlled trial aims to reduce ESA use in patients with CAP by optimizing rapid pathogen detection and improving de-escalation rates following negative cultures. The study has two aims:

- Aim 1: To determine the impact of comprehensive pneumococcal UAT and rapid viral molecular diagnostic assays on antimicrobial prescribing for CAP.
  - Hypothesis 1: Comprehensive diagnostic testing will allow fewer days of ESA.
- Aim 2: To compare pharmacist-led de-escalation of empiric antimicrobial therapy following negative cultures to usual care.
  - Hypothesis 2a: Pharmacist-led de-escalation will reduce the number of days of ESA use.
  - Hypothesis 2b: Pharmacist-led de-escalation will be more effective when physicians order UAT/rapid viral testing.

The study proposal was designed following the recommendations of the Standard Protocol Items: Recommendations for Interventional Trials (SPIRIT) and their checklist is included as an Additional file [15].

### Trial design

We will perform a pragmatic, multicenter 2 × 2 factorial cluster-randomized controlled trial with four arms: rapid diagnostic testing, pharmacist-led de-escalation, rapid diagnostic testing + pharmacist-led de-escalation, and usual care (Table 1).

**Table 1:** Two-by-two factorial cluster-randomized controlled trial with four arms

		Pharmacist-led de-escalation	
		Yes	No
Rapid diagnostic testing	Yes	Rapid diagnostic testing + pharmacist-led de-escalation (3 hospitals)	Rapid testing (3 hospitals)
	No	Pharmacist-led de-escalation (3 hospitals)	Usual care (3 hospitals)

We hypothesize there will be an interaction between the two interventions as the diagnostic tests should make it easier for the pharmacist to recommend to clinicians to de-escalate after 48 h. The factorial design allows for comparing the two interventions in combination and individually, against the standard of care. All interventions will be performed at the hospital level to minimize crossover between study arms and allow for consistent care. We will use a cluster-randomized design to minimize cross-contamination and improve the efficiency of intervention administration.

## Methods: participants, interventions, and outcomes

### Study setting

The Cleveland Clinic is an integrated health system with over 51,000 clinicians and 6000 beds, consisting of 11 regional hospitals in Ohio and 5 in Florida. We identified 12 hospitals (2 large academic medical centers and 10 community hospitals) within the Cleveland Clinic Health System (CCHS) for participation based on the number of annual CAP admissions, teaching status, staffing by the Department of Hospital Medicine, antimicrobial stewardship programs, and hospital size.

### Participating sites

Enrolling sites will include ten hospitals in Northeast Ohio (Cleveland Clinic Main Campus, Fairview Hospital, Hillcrest Hospital, Marymount Hospital, Akron General Hospital, Avon Hospital, Euclid Hospital, Lutheran Hospital, Medina Hospital, and South Pointe Hospital) and two from Florida (Weston and Indian River Hospital) (Table 2). Hospitals will be divided into 3 strata based on teaching status, size of the hospital, and number of CAP patients with each stratum containing 4 hospitals. The hospitals will then be randomized to each of the 4 trial arms with an effective sample size of 3 hospitals in each arm.

**Table 2:** Enrolling hospital sites by bed size and teaching status

Hospital	Number of beds	Residency program
Cleveland Clinic Main Campus	1167	Yes
Fairview General Hospital	488	Yes
Hillcrest Hospital	341	No
Marymount Hospital	195	No
Akron General	532	Yes
Avon Hospital	126	No
Euclid Hospital	110	No
Lutheran Hospital	119	Yes
Medina Hospital	148	No
South Pointe Hospital	167	Yes
Cleveland Clinic Florida, Weston Hospital	230	Yes
Indian River Hospital	332	No

### Eligibility criteria

The inclusion criteria for patient records are as follows:

1. Men or women aged 18 years or older
2. Admitted to a participating (i.e., enrolled and randomized) hospital
3. Admitting diagnosis of pneumonia

The exclusion criteria are as follows:

1. Admission to the intensive care unit (ICU) within 24 h of hospital admission
2. Comfort care measures only
3. Cystic fibrosis (ICD10 codes present on admission)
4. Discharged from an acute care hospital in the past week
5. Patients not eligible for empiric therapy due to a known pathogen (any positive blood or respiratory cultures in the 72 h prior to admission)

### Study recruitment and participant identification

All CCHS hospitals utilize the same Electronic Medical Record (EMR) system which includes an EMR-based clinical decision support system (CDSS). When the emergency department (ED) provider admits a patient age 18 years or older to a medical floor (i.e., non-ICU bed) with an admitting diagnosis of pneumonia, the pneumonia admission order set is triggered. If the physician is in a hospital randomized to the rapid diagnostic testing arm, a CDSS-based alert will be generated in real time, and the form will append orders for diagnostic testing. Initially, this will consist of urinary antigen testing for Streptococcus, but other diagnostic tests may be added later, depending on availability and local policies. For physicians at a hospital randomized to the control condition, ordering will proceed as usual (standard-of-care). They may order any diagnostic test, but none are suggested or appended. Another algorithm will help identify study patients who have negative culture results (blood and/or respiratory) for greater than 48 h and generate a list for the antimicrobial stewardship pharmacist, who will be a member of the study team. The alerts will be audited by the pharmacist daily on weekdays at a centralized location. In clinically stable patients from hospitals randomized to the de-escalation arm, the pharmacist will communicate their recommendations for de-escalation to the clinical providers via secure electronic medical record chat, phone call, or page.

### Who will take informed consent?

The study is a low-risk quality-improvement initiative performed at the hospital level, making it impractical for patients to opt out of the study. We have obtained approval from the Cleveland Clinic Institutional Review Board for a waiver of informed consent. The interventions are considered minimal risk because physicians can override the orders for rapid diagnostic testing and pharmacist recommendations for de-escalation, with final decisions depending on the individual physician’s judgment. All research will be conducted in accordance with the Declaration of Helsinki and the guidelines for Good Clinical Practice.

### Interventions

The study will evaluate (a) routine use of rapid diagnostic testing at the time of admission, (b) pharmacist-led de-escalation after 48 h for clinically stable patients with negative cultures, and c) the interaction between the two.

### Delivery of interventions

#### Rapid diagnostic testing

Eligible patients at hospitals randomized to this arm will undergo testing for viral pathogens (from November to April) and pneumococcal UAT testing. Legionella UAT was not selected as a diagnostic test as Legionella accounts for ~ 1.5% of CAP. For non-ICU admitted patients with a pneumonia diagnosis, the CDSS form will append orders for viral and UAT testing in hospitals randomized to the intervention. Providers in the intervention group will see pre-populated orders for rapid diagnostic testing but can cancel one or both orders if they so choose.

#### Pharmacist-led de-escalation

Another algorithm will identify CAP patients meeting study criteria with negative culture results for more than 48 h, generating a list for the clinical pharmacist. The pharmacist will audit the daily alerts on weekdays at a centralized location and assess patient clinical stability using the 2019 ATS/IDSA criteria for clinical stability:

A. Resolved vital sign abnormalities (temperature ≤ 37.8 °C, heart rate ≤ 100 beats/min, arterial oxygen saturation ≥ 90% or pO_2_ ≥ 60 mmHg on room air, systolic blood pressure ≥ 90 mmHg, and respiratory rate ≤ 24 breaths/min),
B. Normal mental status (as deemed by the clinician), and
C. Ability to maintain oral intake [6].

For clinically stable patients, the pharmacist will recommend antimicrobial de-escalation to clinical providers via secure electronic medical record chat, phone call, or page. De-escalation in this context means stopping all ESA agents (anti-MRSA/anti-pseudomonal) but continuing other antibiotics. The pharmacist’s recommendations will be based on a protocol developed by the Cleveland Clinic Health System’s Antimicrobial Stewardship committee, outlining four possible clinical scenarios: (a) positive culture, (b) negative culture, (c) pneumococcal UAT results, and (d) viral test results.

Pharmacist-led de-escalation efforts will be supported by educational initiatives targeting hospitalists, including presentations about the study and audit-and-feedback for adherence to pharmacist recommendations. Site champions at each hospital will ensure intervention fidelity and encourage physician receptiveness to pharmacist messages/calls.

### Control

Control hospital patients will receive usual care, without specific CDSS alerts and pharmacist-led de-escalation efforts.

### Outcomes

The primary outcome is the duration of exposure to ESA therapy defined by the number of days of antibiotic therapy in the first 21 days of admission, following the National Healthcare Safety Network (NHSN) guidelines. ESA therapy for CAP will be defined as drugs that cover MRSA and Pseudomonas and are on Cleveland Clinic formulary (imipenem, meropenem, piperacillin-tazobactam, aztreonam, cefepime, ceftazidime, tobramycin, ceftazidime-avibactam, ceftolozane-tazobactam, meropenem-vaborbactam, imipenem-cilastatin-relebactam, cefiderocol, ceftaroline, tigecycline, eravacycline, amikacin, linezolid, or vancomycin). Any re-escalation of ESA will be counted towards the total duration of therapy. If two ESAs are administered on the same day, that will count as 2 antibiotic days. For antibiotics that are administered more than once daily, we will follow NHSN guidelines counting multiple doses of the same drug as a single antibiotic day. The duration of therapy for patients who receive a portion of their antimicrobial therapy outside the hospital will be calculated based on documented doses in the EHR and expected doses outlined in the discharge medication list, including IV and PO doses as prescribed.

Secondary outcomes:

1. Within 48 h of admission:
  - Viral testing ordered (yes/no): Proportion of patients for whom viral testing was ordered. We will analyze each virus individually as well as all viruses combined (i.e., any viral testing).
  - Proportion of patients testing positive for influenza within 48 h
  - Detection of RSV (yes/no): Proportion of patients testing positive for RSV
  - Detection of viruses/other CAP pathogens (yes/no): Proportion of patients testing positive for each of the viruses/other CAP pathogens
  - Treatment with anti-viral medications (oseltamivir, zanamivir, peramivir, baloxavir, ribavirin, remdesivir, nirmatrelvir, COVID-19 medications) (yes/no)
  - Proportion of patients for whom *S. pneumoniae* urinary antigen test (UAT) is performed.
  - Proportion of patients with a positive pneumococcal UAT.
2. Treatment with antiviral medications (oseltamivir, zanamivir, peramivir, baloxavir, ribavirin, remdesivir, nirmatrelvir, COVID-19 medications) within 21 days
3. De-escalation of ESA within 72 h from admission (yes/no) — Proportion of patients whose ESA (imipenem, meropenem, piperacillin-tazobactam, aztreonam, cefepime, ceftazidime, tobramycin, ceftazidime-avibactam, ceftolozane-tazobactam, meropenem-vaborbactam, imipenem-cilastatin-relebactam, cefiderocol, ceftaroline, tigecycline, eravacycline, amikacin, linezolid, or vancomycin) are de-escalated within 72 h from admission.
4. Re-escalation to ESA after de-escalation (yes/no) within 21 days from admission. Proportion of patients whose antibiotics were de-escalated and that were subsequently re-escalated to ESA (imipenem, meropenem, piperacillin-tazobactam, aztreonam, cefepime, ceftazidime, tobramycin, linezolid or vancomycin).
5. Total duration of any antibacterial antibiotic within 21 days. Total duration of any antibacterial antibiotic treatment up to 21 days, including re-initiation of antibiotics.
6. Fourteen-day mortality. Patient’s vital status will be ascertained from the EHR if available, otherwise from the Ohio State Death Index.
7. Thirty-day mortality.
8. ICU transfer after admission (> 24 h after admission): Proportion of patients transferred to the ICU > 24 h after admission up to 21 days.
9. Healthcare-associated *Clostridioides difficile* infection (CDI) (yes/no) — CDI after 72 h of admission: Proportion of patients with CDI after 72 h of admission until 30 days.
10. Acute kidney injury (AKI) after 48 h within 21 days (yes/no): Proportion of patients with AKI after 48 h of admission. AKI will be defined as an increase in serum creatinine ≥ 0.3 mg/dl within 48 h or an increase > 1.5 times baseline [16].
11. Total inpatient cost (from the hospital’s cost accounting system): From admission to discharge or 21 days, whichever comes first.
12. Hospital length-of-stay (days, hours) — length of stay will be calculated as whole days from the time of admission to the time of discharge.
13. Empyema (yes/no) — after 48 h, within 21 days
14. Thirty-day readmission (yes/no)
15. Infection with a resistant organism in the future (yes/no) within 6 months after discharge. Resistance to CAP therapy will be defined as resistance to either a respiratory quinolone or to both a beta-lactam/3rd generation cephalosporin and a macrolide. Multi-drug resistance will be defined as any CAP bacterial isolate that tests either intermediate (I) or resistant (R) to at least one agent in three or more antimicrobial classes.

### Participant timeline

**Fig. 1:**
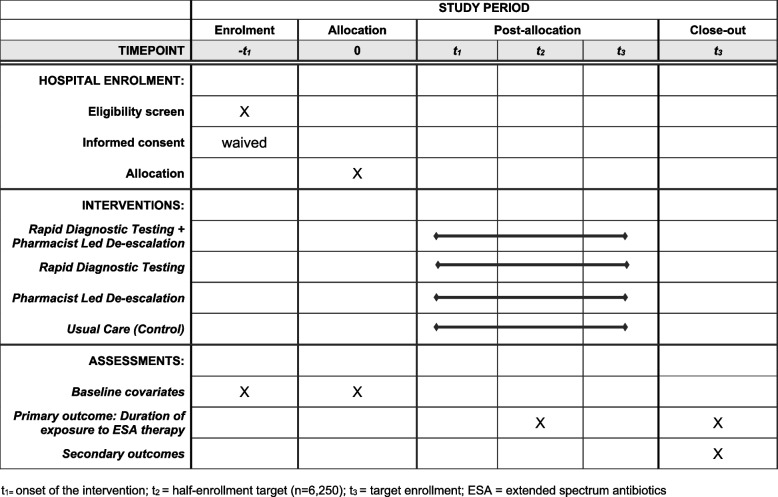
Schedule of enrolment, interventions, and assessments

A schematic diagram highlighting the time schedule of enrolment, interventions, and assessments is presented in Fig. 1.

### Sample size and power analysis

The sample size calculations were based upon the primary outcome of reduction exposure to ESA. For Aim 1, we anticipate an average reduction of 0.3 days (SD = 3.5 days) of exposure to ESAs due to rapid diagnostic testing, based on our preliminary data. To achieve 83% power to detect this effect size at a two-sided alpha of 0.05 and an intra-cluster correlation (ICC) of 0.001, we will randomize 12 hospitals (6 in the rapid diagnostic testing arm and 6 in the control arm) with an average of 1000 patients per hospital (coefficient of variation (COV) = 0.4). This will result in a total sample size of 12,000 patients (6000 patients per arm).

For Aim 2, we will compare the six hospitals implementing pharmacist-led antimicrobial de-escalation and the other six hospitals serving as the control group. De-escalation is expected to reduce exposure to ESAs by 1 day (SD = 3), based on our preliminary data. Assuming a 50% compliance rate in the de-escalation arm, we will have > 99% power to detect this effect size at a two-sided alpha of 0.05 and an ICC of 0.005. We will also have > 99% power to detect a reduction in the rates of CDI from 1 to 0.5% and AKI from 6 to 3%, under the same assumptions. Since testing the interaction is a secondary analysis, power calculation was not performed. All power calculations were conducted using PASS 14 (Kaysville, UT).

### Assignment of interventions: allocation and blinding

Due to the nature of the intervention, participating physicians and hospitals cannot be blinded to their assignment. The principal investigator (PI) and study statisticians will not be blinded to the group allocations. The trial biostatistician will be blinded to the data analyses. The diagnostic testing intervention and the de-escalation intervention, delivered as clinical decision support, will be implemented at the intervention hospitals, limited to participating physicians. To ensure balanced study arms concerning hospital type, we will conduct stratified randomization. Hospitals will be grouped into three strata as described previously. A senior statistician in the Department of Quantitative Health Sciences will program the cluster randomization procedure using STATA 17 MP. The *ralloc* procedure allocates two treatments, called Rx1 and Rx2, to 12 hospitals in three strata using a 2 × 2 factorial design. Blocks of equal frequency allocate treatments labeled “Rx1” and “Rx2” equally in each of the three strata. A random seed was set by the trial statistician and kept hidden. In each stratum, hospitals are arranged in alphabetical order.

### Data collection and quality check

The period of data accrual for each patient will be for 6 months from the time of admission. Data will be extracted from the EMR by the data scientist periodically. Data collection will include patient demographics and characteristics, associated comorbidities, diagnostic and other laboratory tests, antibiotic prescribing data, and the primary and secondary outcomes. A data analyst will extract the data from electronic medical records at both intervention and control hospitals and a statistical programmer will review it for completeness and accuracy. Data will be coded using standardized codes and stored in a secure, password-protected database with regular backups. Any data inconsistencies will be verified by cross-referencing the original medical records. Missing, incorrect, or out-of-range values will be addressed. Routine internal audits of the database will be conducted by the research team.

## Statistical methods

### Data analysis plan

The data analysis will be conducted following the intention-to-treat (ITT) principle which includes 3 rules. The first rule establishes the landmark event for entry into the study, which is the randomization of hospitals. All randomized hospitals will be considered enrolled, and data of eligible patients treated by enrolled hospitals will be analyzed. The second rule establishes patient-level data collection, which is independent of treatment assignment. The third rule defines the pre-specified time period for counting study events, which varies for each outcome.

We will follow the order principle of analyses, which states that analyses involving the primary outcome measure must be preceded by analyses of higher-order outcomes when those outcomes are censored. In our study, all-cause death events are censored. To follow the order principle of analyses, we will compare the all-cause mortality incidence rate within the first 30 days of admission across the four treatment groups. Incidence rate is calculated as the number of all-cause deaths divided by the number of included patients and expressed per 1000 (or 10,000 patients) per 30 days of follow-up. We will calculate the incidence-rate difference between the four treatment groups, using the usual care group as the reference. The four treatment arms are rapid testing and de-escalation, rapid testing only, de-escalation only, and usual care (i.e., neither rapid testing nor de-escalation).

The primary outcome of days on ESA will be analyzed using a linear mixed-effects model, which will include the treatment arm as a fixed effect and a random-intercept at the randomized hospital level. We will adjust for baseline covariates between the intervention and control groups. These covariates include patient age, sex, race, CURB65, Elixhauser index, serum Na, serum glucose, hemoglobin, MRSA nasal swab (yes/no), WBC count, albumin, baseline oxygenation for CAP patients (O_2_ sat off O_2_ or supplemental oxygen delivery device type (vent > bipap > high flow, etc.), max or median respiratory rate on day 1, min SBP on day 1, vasopressors, and comorbidities (chronic pulmonary disease, lymphoma, metastatic cancer, obesity), as well as hospital characteristics in the year prior to study initiation (duration of antibiotics and ESA, AKI, CDI, 14-day mortality, and 30-day mortality, all among CAP patients who would have met study criteria). The primary comparison is to compare the duration of exposure to ESA therapy in the four treatment groups. Interaction between the two interventions will be tested as a secondary analysis. We will consider a two-sided *p*-value < 0.05 statistically significant. As we use a priori stratification (by the type of hospital), we will examine differential intervention effects by each pre-defined stratum. The primary outcome data will be available for every patient since it is recorded in the EHR. In case of missing secondary data, we will assume it is missing at random and a sensitivity analysis will be performed with data imputed via Multiple Imputation by Chained Equations (MICE).

To address the non-adherence limitation, we will conduct two secondary analyses [17]. First, we will perform an as-treated analysis, comparing outcomes among those who receive the treatment versus those who receive control, regardless of randomization. Second, we will calculate complier average causal effect (CACE) estimation, which uses randomization as an instrument to account for unobserved confounding and provides a randomization-respecting estimate [18]. CACE estimates the intention-to-treat effect in the subgroup of participants who always comply with their treatment allocation. In addition, we will use per-protocol analysis, comparing those who comply with their random allocation in the treatment group with all the controls. We will use an inverse probability weighting approach to minimize selection bias [19]. All the secondary analyses will use the framework of mixed-effects (hierarchical, two-level) models as described above for the primary ITT analyses.

All continuous secondary outcomes will be analyzed using the same method as the primary outcome. For each secondary outcome, we will adjust for the rate of that outcome in the 12 months prior to the study. Binary outcomes will be analyzed using generalized linear mixed-effects models. All analyses will be conducted using SAS 9.3 (Cary, NC), R-studio (Boston, MA), and STATA (College Station, TX). Our trial will follow the Consolidated Standards of Reporting Trials guidelines, extended for cluster-randomized controlled trials [20].

### Cost analysis

We will conduct a cost analysis from the health system perspective, including only medical costs related to providing care and excluding costs associated with patient time spent seeking care, caregiver time, transportation, or productivity loss. Total direct medical costs of the hospitalization/inpatient encounter will be collected through the Cleveland Clinic finance system. Costs are grouped into different categories, including inpatient, outpatient, laboratory testing, and pharmacy. For the intervention groups, we will also include the cost of rapid diagnostic testing and time cost of the pharmacists, calculated as hourly salary multiplied by the number of minutes pharmacists spent discussing de-escalation with providers. Due to the right-skewed nature of cost data, we will use bootstrap methods to compare median costs between groups [21]. We will draw 1000 bootstrap samples with replacement from each study group and calculate the mean cost difference and 95% confidence interval. Finally, we will employ a generalized linear mixed-effects model (with a log-link function and gamma distribution) to analyze the interventions' effect on total cost, accounting for differences in baseline patient-level characteristics.

### Data security/storage

Access to the study database will be restricted and require passwords that are only known to relevant study personnel. Devices used to transport data will be CCF-approved encrypted devices with password protection. Data will be maintained for 6 years after the study’s completion, and HIPAA Privacy and Security Rules will be strictly followed.

### Assessment of safety

Unanticipated problems related to the intervention will be logged and reported to the Institutional Review Board (IRB) at the time of continuing renewal. Major deviations will be reported to the IRB upon discovery.

Periodically during the trial, the study team will:

- ▪ Review the research protocol
- ▪ Evaluate the trial’s progress
- ▪ Consider external factors, such as scientific or therapeutic developments that may affect the study’s safety or ethics
- ▪ Review center performance, make recommendations, and assist in resolving problems
- ▪ Protect the safety of study participants
- ▪ Conduct interim analysis, if appropriate
- ▪ Ensure confidentiality of trial data and results of monitoring
- ▪ Address any problems with study conduct, enrollment, sample size, or data collection

Any protocol changes that are deemed significant by the investigator team will be reviewed and approved by the IRB at Cleveland Clinic. This will also be shared by AHRQ. All protocol changes will be documented with version control in place.

### Interim analyses

Safety interim analysis will be conducted every 6 months. The all-cause death incidence rate within the first 30 days of admission is calculated as the number of all-cause deaths divided by the number of eligible patients (according to inclusion and exclusion criteria) and expressed per 1000 (or 10,000 patients) per 30 days of follow-up. We will use the all-cause death incidence rate within the first 30 days of admission calculated during the 1-year period before the study onset as a baseline estimation. We will calculate incidence-rate ratios and incidence-rate differences between the four treatment groups, using the usual care group as the reference. The four treatment arms are rapid testing and de-escalation, rapid testing only, de-escalation only, and usual care (neither rapid testing nor de-escalation). If the all-cause death incidence-rate ratio or incidence-rate difference between the usual care group and any of the three intervention groups is statistically significant at a *p*-value < 0.05, the study’s Steering Committee will be notified.

Futility interim analysis will be conducted after half the data have been collected (patient *n* = 6250). At that time, we will calculate conditional power: the probability of statistical significance at the study’s completion given the date obtained so far. We will calculate the futility index as 1 − conditional power. The study will be stopped if the futility index is above 0.8 (conditional power falls below 0.2). To calculate conditional power, we will calculate current (at *n* ≥ 6250) z-statistic, using the PASS Probability Calculator. PASS 2022 implemented conditional power calculation using Jennison and Turnbull (year 2000; pages 205–208), the general upper one-sided conditional power at stage k for rejecting a null hypothesis about a parameter θ at the end of the study, given the observed test statistic, *Z*_*k*_.

### Dissemination plans

The project’s design emphasizes the intention and plan to utilize the knowledge and products gained to improve patient care. The trial was registered at ClinicalTrials.gov before enrolling the first patient. As the study progresses, we will update the trial progress and recruitment status (not yet enrolling–enrolling–enrollment completed). To encourage the translation of the study’s results into practice, we will disseminate the findings widely through conference presentations (e.g., CHEST annual conference, *IDWeek*, Society of Hospital Medicine) and peer-reviewed publications. The large scale and pragmatic design of our study make it unique. The knowledge obtained from this study will likely inform CAP guidelines on the potential of rapid molecular diagnostic assays to reduce ESA use and the safety and efficacy of antimicrobial de-escalation following negative cultures. Additionally, study team members will collaborate with their respective professional societies. Dr. Klompas, who has served on various pneumonia guideline panels, will help disseminate the findings at the Infectious Disease Society of America (IDSA) and Society for Healthcare Epidemiology of America (SHEA). Dr. Haessler, a board of trustees member for SHEA, will assist in disseminating the findings at SHEA meetings and conferences. Authorship on any future publications utilizing trial data will be evaluated individually, contingent on the contributor’s involvement following ICJME guidelines.

## Discussion

Our large, multicenter 2 × 2 factorial cluster-randomized controlled trial will be the first to assess two strategies for reducing ESA use in patients with CAP: (a) routine rapid diagnostic testing at admission and (b) pharmacist-led de-escalation after 48 h for clinically stable patients with negative cultures. The size and randomized design of our study will allow us to establish causality between the interventions and the duration of antimicrobial exposure. The resultant reduction in antibiotic exposure should allow us to test the relationship between ESA exposure and adverse outcomes such as CDI and AKI. By demonstrating the safety of de-escalation and the harms of prolonged ESA exposure, we hope to provide strong evidence supporting the ATS/IDSA recommendations on antimicrobial de-escalation and diagnostic testing, thereby promoting their adoption by clinicians.

The study has several limitations, addressed by its design. First, physicians may resist pharmacist recommendations for de-escalation. To mitigate this, we have secured support from senior leadership at the hospital and departmental levels. Educational efforts will target all participating physicians in the intervention hospitals, and those with low adherence levels will receive feedback and targeted education from the site champion. Second, capturing secondary events, particularly those occurring after discharge, may prove difficult. To address this, we will ascertain patients' vital status from the EHR, Ohio State Death Index, or other existing registries in CCHS. Lastly, uncertainties surrounding the COVID-19 pandemic’s impact on our study exist. If the pandemic intensifies again during the study, the circulation of influenza/RSV may be affected. Even if the pandemic remains under control, infection control measures may persist (e.g., masking and social distancing during respiratory virus season, and improved ventilation). To accommodate these potential changes, our trial will include at least the two most prevalent causes of CAP, and we may consider adding other rapid diagnostic tests (e.g., atypical CAP pathogens and other viruses) if economically feasible, such as when available in a single panel at no increased cost.

In conclusion, our pragmatic cluster-randomized trial offers a rigorous design to test two approaches to reducing the use of broad-spectrum antibiotics in patients with CAP. This will enable us to establish causality and determine whether ESAs can be safely de-escalated in stable patients.

## Trial status

As the manuscript is finalized, the trial is currently in the process of enlisting participants. The enrollment period commenced on November 1, 2022. This protocol represents version 1, with a date of March 31, 2023. The trial’s conclusion is anticipated in November 2025. World Health Organization (WHO) Trial Registration Data Set: All items from the WHO Trial Registration Data Set can be found within the protocol.

### Supplementary Information

**Additional file 1.**

## Data Availability

The complete study protocol can be accessed in this publication as well as in the ClinicalTrials.gov record. Upon completion, the study outcomes will be presented on ClinicalTrials.gov and disseminated via the publication of the findings. De-identified participant data, subject to compliance with organizational policies; local institutional review board rules; local, state, and federal laws and regulations, including the HIPAA Privacy Rule; and the AHRQ data sharing policy.
